# Morphological and molecular evidence support the inclusion of *Mycetia hainanensis* in *Mouretia* (Rubiaceae)

**DOI:** 10.3897/phytokeys.270.180324

**Published:** 2026-02-05

**Authors:** Dongxian Xu, Li Li, Buyun Zhang, Zhuqiu Song

**Affiliations:** 1 Guangdong Academy of Forestry, Guangzhou 510520, China Chinese Academy of Sciences Guangzhou China; 2 Key Laboratory of National Forestry and Grassland Administration on Plant Conservation and Utilization in Southern China, South China Botanical Garden, Chinese Academy of Sciences, Guangzhou 510650, China Guangdong Academy of Forestry Guangzhou China

**Keywords:** Argostemmateae, *
Mouretia
*, *
Mycetia
*, phylogeny, species tree, taxonomy

## Abstract

A key diagnostic feature of the genus *Mycetia* is its berry-like fruits, which distinguish it from its capsular-fruited relatives in the tribe Argostemmateae. However, the fruit character of *Mycetia hainanensis* H.S.Lo has long remained uncertain, as the species was previously known only from flowering specimens. Our rediscovery of this species at the type locality has now resolved this uncertainty. We found that *M. hainanensis* produces fleshy capsules that open via an apical operculum and flowers with a corolla tube shorter than 4 mm long. This finding first prompted a morphological re-evaluation, which indicated that the species aligns more closely with the genus *Mouretia*. Subsequent molecular phylogenetic analyses of plastid and nuclear DNA data robustly supported this hypothesis, placing the species within *Mouretia*. Given the congruent evidence from both morphology and molecular phylogeny, we propose the transfer of *M. hainanensis* to *Mouretia*, along with a complete morphological description. As part of this revision, we also provide keys to all genera in the Argostemmateae and to all species in *Mouretia*, along with a distribution map of the genus.

## Introduction

Argostemmateae Bremek. ex Verdc. is a medium-sized tribe in the subfamily Rubioideae of Rubiaceae with a predominantly Asian distribution. The tribe comprises six genera and roughly 255 accepted species: *Argostemma* Wall. (179 Asian and two African species), *Clarkella* Hook.f. (monotypic), *Leptomischus* Drake (11 species), *Mouretia* Pit. (five species), *Mycetia* Reinw. (55 species), and *Neohymenopogon* Bennet (three species) (Razafimandimbison and Rydin 2024; Hassler 2025). The highest species richness is found in the Malesian region, which harbors more than half of all species (ca. 142 species), although these are restricted to *Argostemma* and *Mycetia*. Indochina also exhibits high diversity, hosting all six genera and about 93 species. In China, approximately 37 species within six genera have been recorded (Chen et al. 2011; Yan et al. 2016; Song and Xu 2016; Liu et al. 2022; Quan et al. 2024; Hassler 2025).

Morphologically, Argostemmateae species have hermaphroditic flowers, bilocular ovaries, fruits crowned by persistent calyx lobes, and numerous dust-like seeds (Razafimandimbison and Rydin 2019). Within the tribe, *Mycetia* is distinguished by its indehiscent, berry-like fruits (Song et al. 2025). At maturity, these fruits become white, enlarged, and spongy due to the formation of internal air spaces—a character hypothesized as an adaptation for avian dispersal (Puff et al. 2005). In contrast, species of the other five genera produce capsular fruits, which dehisce either septicidally through a beak in *Neohymenopogon* or via an apical operculum in *Argostemma*, *Leptomischus*, and *Mouretia*. These latter three genera can be further distinguished from one another based on floral characters, particularly the length of the corolla tube.

*Mycetia hainanensis* H.S.Lo is an imperfectly known species endemic to Hainan Province, China (Lo 1999; Chen et al. 2011). This species was originally described by Lo (1991) based on seven flowering specimens. In the protologue, the author stated that it differs from *Mycetia hirta* Hutch. by its short internodes (3–6 mm long) and calyx lobes without stipitate glands. It is also described as a subshrub, 10–25 cm high, bearing capitate inflorescences that are solitary or in groups of three and consist of white subsessile flowers. This suite of characters—plant height, internode length, inflorescence type, flower color, subsessile flowers, and eglandular calyx lobes—calls the generic placement of *Mycetia hainanensis* into question. This is because *Mycetia* typically comprises taller plants with longer internodes, comparatively looser inflorescences, yellow flowers, distinct floral pedicels, and glandular calyx lobes, although some variation in these characters occurs within the genus (Deb 1986; Puff et al. 2005; Chen et al. 2011; our own observations). Furthermore, the fruit character, a key diagnostic character of *Mycetia*, has remained unknown for this species to date (Lo 1991, 1999; Gao et al. 2005; Chen et al. 2011, 2015).

In this study, we aim to elucidate the generic placement of *Mycetia hainanensis* through an integrated analysis of molecular and morphological data and to provide, for the first time, a complete description of this species.

## Materials and methods

### Morphological studies

We examined the holotype and all six paratypes of *Mycetia hainanensis*, along with all available specimens of congeneric species deposited in the following herbaria: A, BKF, BISH, BM, BO, CANT, E, GH, GXMI, HITBC, IBK, IBSC, K, L, NY, MICH, MO, US, PE, SING, SYS, and U. Herbarium codes follow Index Herbariorum (Thiers 2025). In recent years, we conducted multiple field surveys at the type locality (Jianfengling, Hainan Province) to observe the floral and fruit characteristics of the species. Additionally, we performed morphological examinations of several genera related to *Mycetia*, including *Argostemma*, *Clarkella*, *Leptomischus*, *Mouretia*, and *Neohymenopogon*.

### Phylogenetic analyses

We conducted a plastid phylogenetic analysis of the tribe Argostemmateae based on a concatenated dataset of five plastid DNA regions (*atpB-rbcL*, *ndhF*, *rbcL*, *rps16*, and *trnTF*) from 42 species representing all six genera of the tribe and two outgroups from other tribes. Among these, two individuals of *Mycetia hainanensis* were newly sequenced for this study; all other sequences were derived from Song et al. (2025; Suppl. materials 1, 3). Procedures for DNA extraction, sequencing, assembly, annotation, alignment, and trimming followed those described in that study. The Maximum likelihood (ML) phylogenetic tree was reconstructed using IQ-TREE v3.0.1 (Nguyen et al. 2015). Branch support was assessed using the Shimodaira–Hasegawa approximate likelihood ratio test (SH-aLRT) and ultrafast bootstrap (UFBoot), each with 1,000 replicates. Clades were considered strongly supported if they received SH-aLRT ≥ 80% and UFBoot ≥ 95%.

To provide an independent assessment of phylogenetic relationships within a broader phylogenetic context, we reconstructed a nuclear phylogeny of the Spermacoceae alliance (including Argostemmateae and ten related tribes) based on a target-capture dataset of 353 nuclear genes (Suppl. material 4), following the framework established by Thureborn et al. (2022). Taxon sampling covered five genera of Argostemmateae (excluding *Leptomischus*, for which no material was available) and one representative from each of the ten other related tribes. The ingroup included two species of *Mycetia* (*M. bracteata* and *M. hainanensis*), with two samples from the tribe Coussareeae serving as outgroups (Suppl. material 2). Nuclear genes were captured using HybPiper v2.3.2 (Johnson et al. 2016) with the Angiosperm353 target sequence file (Johnson et al. 2019). The dataset was filtered by removing genes shorter than 70% of the average length, genes present in fewer than 15 samples, and samples with fewer than 200 genes. Individual gene trees were obtained under the same ML approach applied in the plastid analysis. These gene trees were then used to infer the species tree using ASTRAL-III v5.7.8 (Zhang et al. 2018). Branch support was quantified using local posterior probabilities (LPP), and clades with LPP > 0.9 were considered strongly supported (Sayyari and Mirarab 2016).

## Results

### Morphological examination

**Figure 1.**
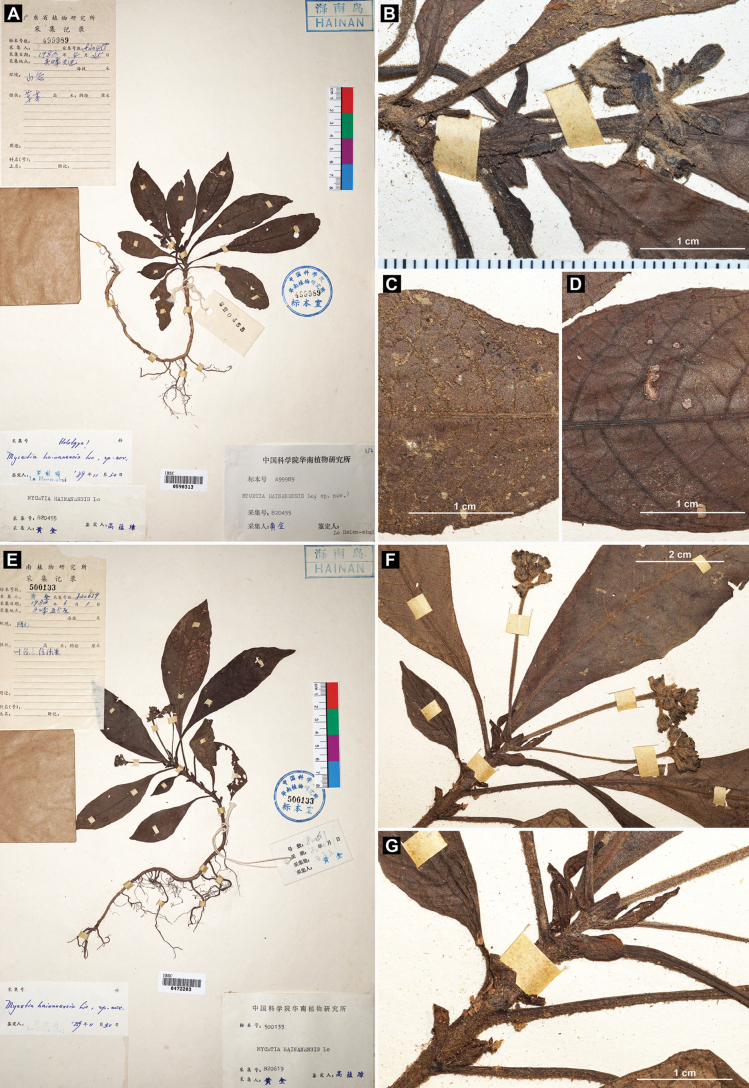
Type specimens of *Mycetia hainanensis* H.S.Lo. **A–D**. Holotype (Q. Huang 820455, IBSC0590313); **E–G**. Paratype (Q. Huang 820619, IBSC0472203). **B**. Inflorescence; **C**. Leaf in abaxial view; **D**. Leaf in adaxial view; **F**. Inflorescence; **G**. Stipules.

**Figure 2.**
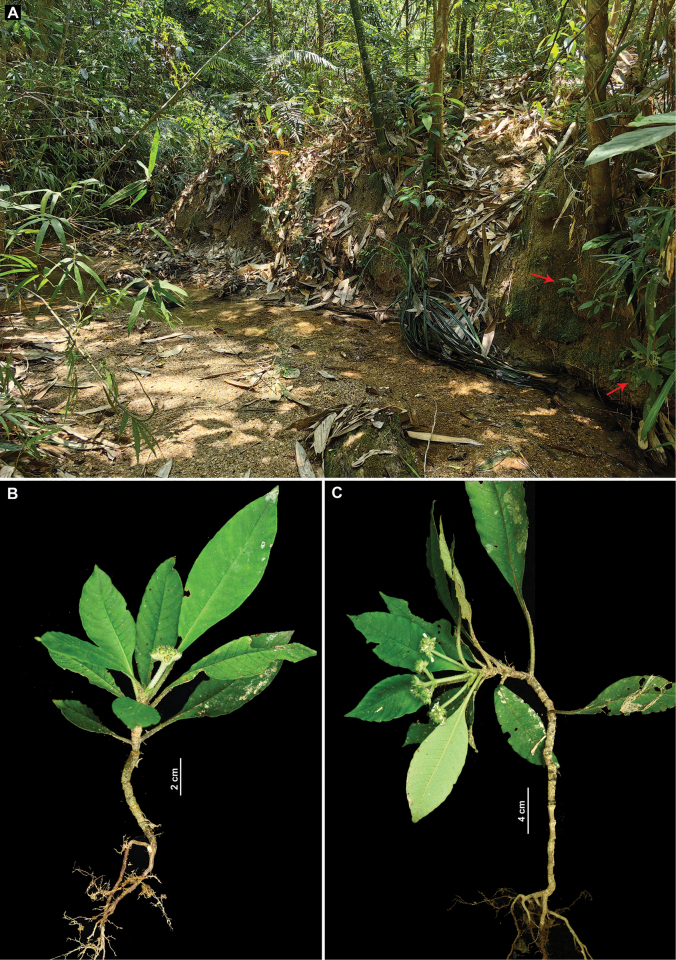
*Mycetia hainanensis* H.S.Lo. **A**. Habitat (red arrows indicate individual plants); **B, C**. Individuals with different inflorescence morphology.

**Figure 3.**
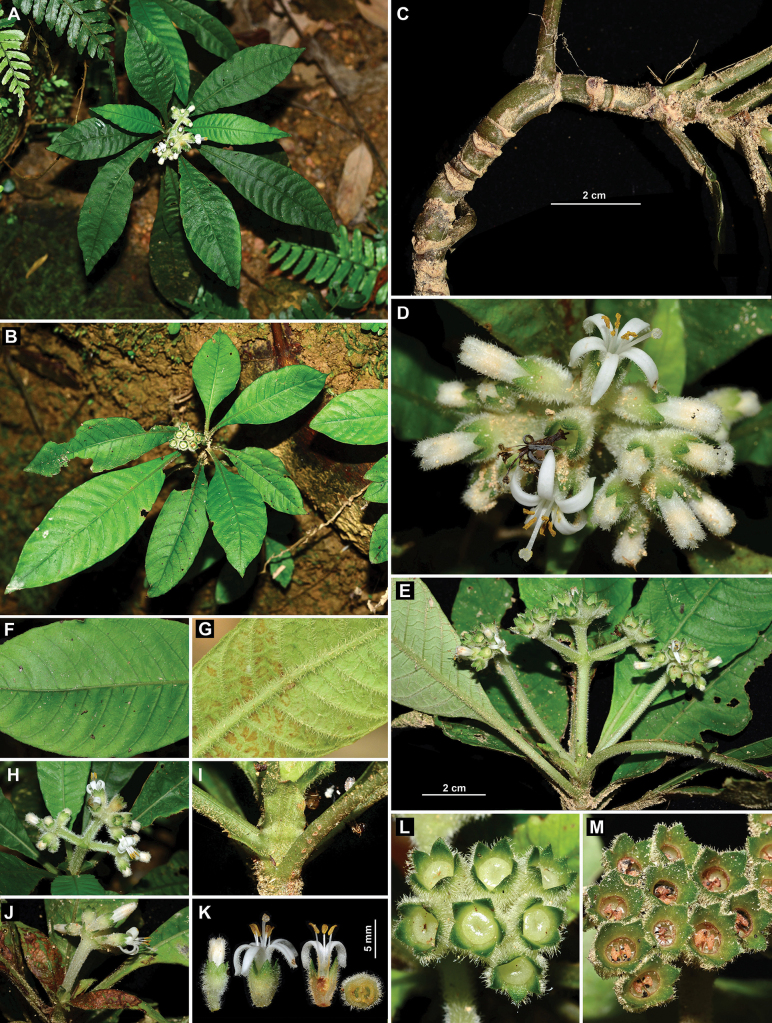
*Mycetia hainanensis* H.S.Lo. **A**. Flowering individual; **B**. Fruiting individual; **C**. Stem with short internodes; **D**. Inflorescence with opened and unopened flowers; **E**. Inflorescence; **F**. Leaf in adaxial view; **G**. Leaf in abaxial view; **H**. Inflorescence; **I**. Stipule; **J**. Inflorescence; **K**. Unopened and opened flowers, showing longitudinal and transverse sections; **L**. Part of immature infructescence, showing immature fruits; **M**. Part of infructescence, showing mature fruits. Photos by Zhuqiu Song (**A–L**) and Li Li (**M**).

Our examination of the holotype (Fig. 1A) and six paratypes (one illustrated in Fig. 1E) of *Mycetia hainanensis* showed that these specimens are small herbs bearing unopened flowers or young fruits. The flowers are densely clustered into capitate or subcapitate inflorescences on long peduncles (Fig. 1B), occasionally with three such inflorescences grouped on a short common peduncle (Fig. 1F). The calyx and corolla are densely hirtellous externally (Fig. 1B, F). The stipules are ovate to elliptic with an obtuse apex (Fig. 1B, F). The leaves are glabrous adaxially (Fig. 1D) but villous to villosulous abaxially (Fig. 1C). At the type locality of the species (Jianfengling, Hainan), we successfully found many living individuals (Fig. 2A), which exhibit characteristics fully consistent with the type material, including plant height (Fig. 2B, C), internode length (Fig. 3C), stipule shape (Fig. 3I), leaf indumentum (Fig. 3F, G), inflorescence type (Fig. 3E, H, J), flower color (Fig. 3D, E, H, J), subsessile flowers (Fig. 3K), and the hirtellous (Fig. 3K) and eglandular (Fig. 3K) nature of the calyx. We therefore confirm that the living individuals collected in the field are conspecific with the aforementioned herbarium specimens of *Mycetia hainanensis*.

However, our field investigations revealed for the first time that *Mycetia hainanensis* produces fleshy capsular fruits that dehisce via an apical operculum at maturity (Fig. 3M), contrasting with the indehiscent, berry-like fruits of *Mycetia*. Morphological comparison among the six related genera of the tribe Argostemmateae indicated that *Mycetia hainanensis* aligns more closely with *Mouretia* (Key 1). It can be distinguished from all five known *Mouretia* species by its homostylous flowers with stamens fully exserted beyond the corolla tube and filaments 3.5–3.8 mm long (Key 2).

### Phylogenetic placement

**Figure 4.**
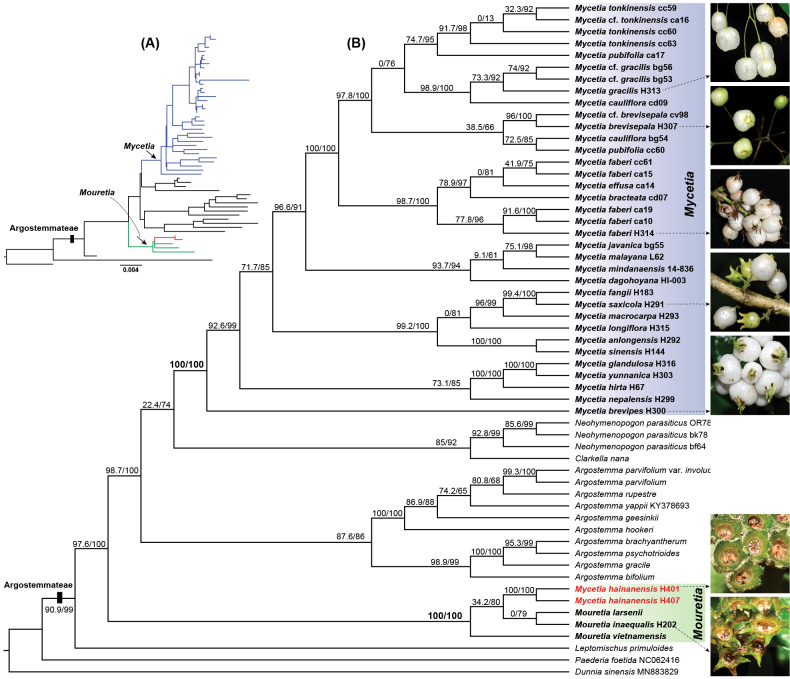
Maximum likelihood phylogeny of the tribe Argostemmateae inferred from a concatenated dataset of five plastid markers (*atpB-rbcL*, *ndhF*, *rbcL*, *rps16*, and *trnTF*) using IQ-TREE v3.0.1. **A**. Phylogram with tip labels omitted, where blue lines show species of *Mycetia*, green lines show species of *Mouretia*, and red lines indicate *Mycetia hainanensis*; **B**. Cladogram with transformed branches, showing photographs of mature fruits of selected representative species. Numbers at branches indicate support values (SH-aLRT/UFBoot).

**Figure 5.**
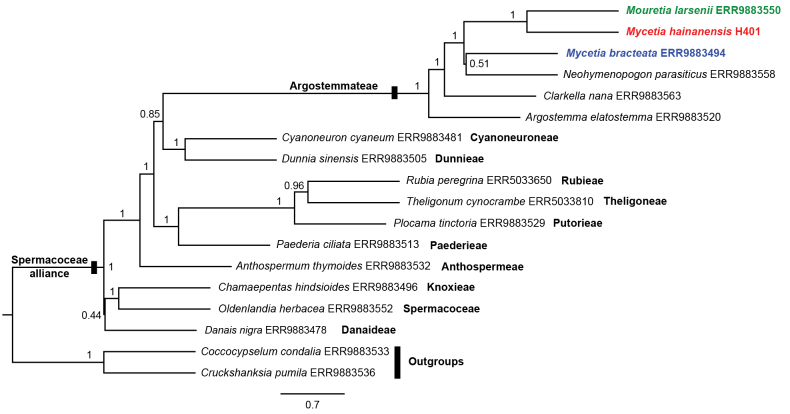
Species tree of the Spermacoceae alliance (including Argostemmateae and ten related tribes) based on 315 nuclear gene trees using ASTRAL-III v5.7.8. Numbers near branches are local posterior probabilities (LPP).

Maximum likelihood (ML) analysis of the concatenated plastid dataset yielded a well-supported plastid tree (Fig. 4). The tribe Argostemmateae was strongly supported as a monophyletic group (SH-aLRT = 90.9%, UFBoot = 99%). Within the tribe, *Mycetia* was not monophyletic. *Mycetia hainanensis* was nested within *Mouretia* with high support (SH-aLRT = 100%, UFBoot = 100%), whereas the remaining 23 *Mycetia* species formed a distinct, well-supported clade (SH-aLRT = 100%, UFBoot = 100%). Coalescent-based analysis of 315 nuclear genes yielded a well-supported species tree with generally high support (Fig. 5). Again, Argostemmateae was monophyletic (local posterior probability, LLP = 1), although the generic relationships differed from those in the plastid tree. *Mycetia hainanensis* was resolved as sister to *Mouretia larsenii* Tange (LLP = 1), while *Mycetia bracteata* formed a weak clade with *Neohymenopogon parasiticus* (Wall.) Bennet (LLP = 0.51). Collectively, both plastid and nuclear phylogenetic analyses support a closer relationship of *Mycetia hainanensis* with *Mouretia* than with *Mycetia*.

## Discussion

Within the tribe Argostemmateae, fruit morphology is classified into three main types: berries (exclusively found in *Mycetia*), dry capsules (in *Clarkella* and *Neohymenopogon*), and fleshy capsules (present in *Argostemma*, *Leptomischus*, and *Mouretia*) (Rydin et al. 2009; Chen et al. 2011; Razafimandimbison and Rydin 2019; Thureborn et al. 2022). *Mycetia hainanensis* produces fleshy capsules that dehisce via an apical operculum at maturity (Fig. 3M), thus providing definitive evidence for its previous misplacement in the genus *Mycetia*.

In terms of inflorescence and floral morphology, the genera within the tribe Argostemmateae exhibit further diagnostic differences. *Neohymenopogon* is distinguished by the presence of showy bracts subtending the inflorescence, a feature considered unique within the tribe (Rydin et al. 2009). *Argostemma* typically bears rotate flowers with a very short to nearly absent corolla tube and adnate anthers (Bremer 1987; Quan et al. 2025). In contrast, the other five genera produce tubular to salverform flowers with a distinct corolla tube and free anthers (Chen et al. 2011). Specifically, *Clarkella*, *Leptomischus*, and *Neohymenopogon* possess salverform to salverform-funnelform flowers characterized by a prolonged corolla tube, usually 13–60 mm long and rarely 6–7 mm long (Chen et al. 2011; Wu et al. 2020; Anh et al. 2021a; Nuraliev et al. 2022; Quan et al. 2024). In comparison, *Mouretia* exhibits tubular flowers with a significantly shorter corolla tube of 2.5–5 mm (Tange 1997; Anh et al. 2021b). Furthermore, flowers of *Leptomischus* and *Mouretia* are often sessile and arranged in subcapitate cymes (Tange 1997; Anh et al. 2021b; Quan et al. 2024). *Mycetia hainanensis* produces subcapitate cymes bearing flowers with a 3.5–3.8 mm long corolla tube and free anthers, thus supporting its closer morphological affinity to the genus *Mouretia*.

**Figure 6.**
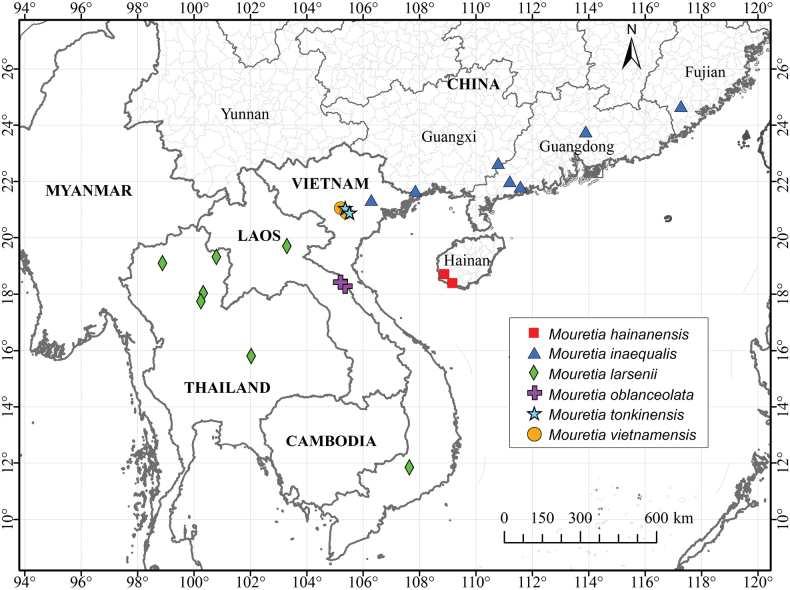
Distribution map of the genus *Mouretia*.

Based on comprehensive morphological and molecular phylogenetic analyses, our results unequivocally support the transfer of *Mycetia hainanensis* from *Mycetia* to *Mouretia* (Fig. 4), thereby resolving the long-standing taxonomic uncertainty regarding the generic placement of this species. Prior to this study, five species were recognized in the genus *Mouretia* (Tange 1997; Anh et al. 2021b; Quang et al. 2023), all distributed from South China to Indochina (Fig. 6). The newly transferred species, *Mouretia hainanensis*, is distinguished within the genus by its homostylous flowers and fully exserted stamens (Fig. 3D, K). Additional diagnostic characters include oblanceolate to narrowly elliptic leaves, ovate to elliptic stipules, terminal inflorescences with a peduncle 1.5–4 cm long, narrowly oblong corolla lobes, and filaments 3.5–3.8 mm long, which collectively aid in the identification of this taxon.

The emergence of homostyly in *Mouretia hainanensis* provides a diagnostic character distinguishing it from all other heterostylous congeners. The presence of homostyly in an otherwise heterostylous genus is noteworthy, as it represents the sole character inconsistent with the proposed transfer. Heterostyly is considered ancestral within the Spermacoceae alliance, with reversals to homostyly occurring primarily in Anthospermeae and Rubieae, as well as in Argostemmateae (e.g., in *Argostemma* and *Neohymenopogon*) (Ferrero et al. 2012). One hypothesis suggests that the loss of heterostyly may be associated with shifts from tropical to non-tropical regions, although this correlation remains unverified (Ferrero et al. 2012). Another hypothesis is that heterostylous species tend to be rare or absent on oceanic islands (Watanabe and Sugawara 2015). *Mouretia hainanensis* occurs on Hainan, which, despite its proximity to the mainland, is an island, whereas all other heterostylous *Mouretia* species are distributed on the mainland. This biogeographic context may explain the occurrence of this anomalous trait in *M. hainanensis*.

### Taxonomic treatment

#### Key 1: Key to the genera of the tribe Argostemmateae

**Table:** 

1	Shrubs, rarely subshrubs; branches with conspicuous, soft, spongy, pale, corky bark; inflorescence often with sessile or stalked glands; fruits berry-like when mature, white, spongy, indehiscent	***Mycetia* Reinw**.
–	Shrubs or herbs; branches usually solid, hard, not spongy; inflorescence often without glands; fruits capsular, dehiscent	**2**
2	Shrubs; inflorescences with enlarged petaloid bracts; fruits septicidally dehiscent through beak or sometimes splitting deeply into 2 valves	***Neohymenopogon* Bennet**
–	Herbs; inflorescences without enlarged petaloid bracts; fruits dehiscent through apical lids formed from disk portion	**3**
3	Corolla broadly rotate or campanulate, without a clear corolla tube; anthers large, mostly connate into a tube around the style, rarely free, exserted, usually opening through an operculum, rarely with longitudinal slit; ovules and seeds borne on peltate placentas attached near top of septum	***Argostemma* Wall**.
–	Corolla tubular-funnelform to salverform, with a clear corolla tube; anthers small, free, usually included, rarely exserted, opening with longitudinal slit; ovules and seeds borne on peltate placentas attached near or below the middle of the septum	**4**
4	Erect herbs with well-developed tubers; flowers homostylous; calyx clearly enlarged during fruiting period	***Clarkella* Hook.f**.
–	Herbs, ascending to procumbent, usually lower nodes with adventitious roots, without well-developed tubers; flowers usually heterodistylous, rarely homostylous; calyx not enlarged during fruiting period	**5**
5	Corolla tube usually longer than 14 mm, rarely 6–7 mm long; ovules and seeds borne on stipitate placentas near base of septum	***Leptomischus* Drake**
–	Corolla tube 2.5–5 mm long; ovules and seeds borne on peltate placentas near middle of septum	***Mouretia* Pit**.

#### Key 2: Key to the species of the genus *Mouretia*

**Table:** 

1	Inflorescence star-shaped cymose, with fused hypanthia; the arms with 4 rows of flowers; style hairy	***Mouretia larsenii* Tange**
–	Inflorescence umbrella-like or head-like cymose, with free hypanthia; style glabrous	**2**
2	Flowers homostylous; corolla lobes narrowly oblong; stamens fully exserted beyond the corolla tube, with 3.5–3.8 mm long filaments	***Mouretia hainanensis* (H.S.Lo) Z.Q.Song & D.X.Xu**
–	Flowers heterodistylous; corolla lobes triangular or ovate; stamens inserted in the corolla tube, with 0.1–2.2 mm long filaments	**3**
3	Leaves anisophyllous; peduncle 2–5 mm long	***Mouretia inaequalis* (H.S.Lo) Tange**
–	Leaves isophyllous; peduncle 5–55 mm long	**4**
4	Inflorescence umbrella-like; peduncles 20–55 mm long; stem sub-erect	***Mouretia oblanceolata* L.Wu, K.S.Nguyen, B.H.Quang & T.P.Anh**
–	Inflorescence head-like; peduncles 5–20 mm long; stem procumbent	**5**
5	Inflorescences terminal and solitary; peduncles 10–20 mm long; calyx lobes ovate to obovate; stipules rounded to reniform	***Mouretia tonkinensis* Pitard**
–	Inflorescences axillary and paired at nodes; peduncles 5–10 mm long; calyx lobes triangular; stipules oblong to obovate	***Mouretia vietnamensis* Tange**

##### Mouretia hainanensis

Taxon classificationPlantaeGentianalesRubiaceae

(H.S.Lo) Z.Q.Song & D.X.Xu comb. nov.

332A9030-D165-5C01-A863-247CA5678AA9

urn:lsid:ipni.org:names:77376422-1

Figs 1, 2, 3

###### Basionym.

*Mycetia hainanensis* H.S.Lo, Guihaia 11 (2): 112. 1991.

###### Type.

China • Hainan Province, Ledong County, Jianfeng [Jianfengling], Tianchi, in valley, 25 April 1982, *Q. Huang 820455* (holotype: IBSC0590313!; Fig. 1A).

###### Description.

***Perennial herbs***, 10–25 cm tall, sub-erect to procumbent, terrestrial. ***Stem*** unbranched or little branched, terete, hirtellous or villosulous when young, glabrescent when mature, 4–6 mm in diameter, with usually 3–17 mm long internodes, without straw-yellow and corky bark. ***Leaves*** simple, opposite, generally isophyllous; petiole 1–6 cm, villosulous; blade slightly succulent, chartaceous when dry, adaxially green, abaxially grayish green, oblanceolate or narrowly elliptic, 5–12 × 3–4 cm, adaxially glabrous, abaxially villous to villosulous, base acute to attenuate, apex acute or weakly acuminate; secondary veins 9–12 pairs. ***Stipules*** interpetiolar, persistent, entire, ovate to elliptic, 5–10 × 3.5–6 mm, puberulent outside, obtuse, green, with undulate margins. ***Inflorescences*** terminal, solitary or in groups of three on a short common peduncle, capitate or subcapitate, densely villosulous, pedunculate; peduncles 1.5–4 cm long, villosulous; heads ca. 1.5 × 1.5 cm, with 7–10 flowers; bracts lanceolate to ovate, large at base of peduncles, 3.8–10 × 1.6–1.8 mm, small at base of pedicel, 1.3–2.3 × ca. 1 mm, sometimes apparently reduced. ***Flowers*** bisexual, homostylous, 5-merous, 10–12 mm long, subsessile to sessile, rarely with a 3 mm long pedicel. ***Calyx*** densely hirtellous, campanulate; tube whitish, ca. 3.3 mm high, ca. 3 mm wide; lobes ovate-triangular, 2.2–2.7 × 1.6 mm. ***Corolla aestivation*** valvate. ***Corolla*** white, tubular, outside hirtellous; tube 3.5–3.8 mm long, ca. 1.3 mm wide, inside with a ring of hairs at the middle; lobes narrowly oblong, revolute, ca. 4.5–5 mm × 1 mm. ***Stamens*** inserted near the middle of the corolla tube, exserted; filaments 3.5–3.8 mm long, white; anthers dorsifixed near base, narrowly oblong, 1.6–1.8 × 0.7 mm, yellow, opening by longitudinal slits. ***Ovary*** 2-locular with numerous ovules; disk concave; style filiform, ca. 8.7 mm long, white, glabrous; stigma exserted, capitate, and very shortly 2-lobed, densely papillate. ***Fruits*** capsular, obdeltoid, ca. 4 mm long, crowned by the persistent calyx lobes, dehiscent through an apical lid formed from the disk portion. ***Seeds*** numerous, angular, black, 0.3–0.5 mm in diameter.

###### Phenology.

Flowering from April to May and fruiting from May to July.

###### Distribution and habitat.

*Mouretia hainanensis* is currently known from Ledong County and Sanya City, Hainan Province, China (Fig. 6), and it grows in the understory of moist ravine forests, usually along streams at 650–800 m elevation.

###### Additional specimens examined.

China • Hainan Province, Ledong County, Jianfeng [Jianfengling], in wet environment, 1 June 1982, *Q. Huang 820619* (IBSC0472203!); • Hainan Province, Ledong County, Jianfeng [Jianfengling], by stream, 13 April 1982, *Q. Huang 820036* (IBSC0472204!); • Hainan Province, Ledong County, Jianfengling, in ravine, alt. 800 m, 29 May 1959, *C. F. Wei 122502* (IBSC0472205!); • Hainan Province, Ledong County, Jianfengling, Feishuikeng, in ravine, 9–10 April 1955, *H. Y. Liang 68605* (IBSC0472206!); • Hainan Province, Ledong County, Jianfengling, by stream, alt. 700 m, 1 October 1957, *H. D. Zhang 3517* (IBSC0472207!); • Hainan Province, Ledong County, Jianfengling, in ravine, dense forest, white flowers, 9 April 1988, *B. H. Chen 745* (IBSC0750022!, IBSC0743232!); • Hainan Province, Ledong County, Jianfengling, in ravine, alt. 654 m, 8 May 2025, *Z.Q. Song & D.X. Xu 2025008* (IBSC); • ibid., in ravine, alt. 840 m, 8 May 2025, *Z.Q. Song & D.X. Xu 2025009* (IBSC); • Hainan Province, Ya County [Sanya City], Xiaobaokang [Baogu cun], in ravine, 11 August 1933, *H. Y. Liang 62533* (IBSC0472208!, IBK00100422, photo!).

## Supplementary Material

XML Treatment for Mouretia hainanensis

## Additional information

### Use of AI

No use of AI was reported.

### Author ORCIDs

Dongxian Xu https://orcid.org/0000-0002-7672-8300

Li Li https://orcid.org/0000-0002-2033-6367

Buyun Zhang https://orcid.org/0000-0003-1201-7638

Zhuqiu Song https://orcid.org/0000-0002-7763-2431

### Data availability

All of the data that support the findings of this study are available in the main text or Supplementary Information.

## Supplementary materials

Supplementary material 1 Voucher information for the plastid DNA samples used in this study Data type xlsx https://binary.pensoft.net/file/1533169This dataset is made available under the Open Database License (http://opendatacommons.org/licenses/odbl/1.0/). The Open Database License (ODbL) is a license agreement intended to allow users to freely share, modify, and use this Dataset while maintaining this same freedom for others, provided that the original source and author(s) are credited. Zhuqiu Song

Supplementary material 2 Voucher information for the nuclear DNA samples used in this study Data type xlsx https://binary.pensoft.net/file/1533170This dataset is made available under the Open Database License (http://opendatacommons.org/licenses/odbl/1.0/). The Open Database License (ODbL) is a license agreement intended to allow users to freely share, modify, and use this Dataset while maintaining this same freedom for others, provided that the original source and author(s) are credited. Zhuqiu Song

Supplementary material 3 Concatenated dataset of five plastid DNA regions (*atpB-rbcL*, *ndhF*, *rbcL*, *rps16*, and *trnTF*) used in this study Data type fasta https://binary.pensoft.net/file/1533171This dataset is made available under the Open Database License (http://opendatacommons.org/licenses/odbl/1.0/). The Open Database License (ODbL) is a license agreement intended to allow users to freely share, modify, and use this Dataset while maintaining this same freedom for others, provided that the original source and author(s) are credited. Zhuqiu Song

Supplementary material 4 Dataset of 315 nuclear genes used in this study Data type zip https://binary.pensoft.net/file/1533172This dataset is made available under the Open Database License (http://opendatacommons.org/licenses/odbl/1.0/). The Open Database License (ODbL) is a license agreement intended to allow users to freely share, modify, and use this Dataset while maintaining this same freedom for others, provided that the original source and author(s) are credited. Zhuqiu Song
