# Corrigendum: Xu D, Li L, Zhang B, Song Z (2026) Morphological and molecular evidence support the inclusion of *Mycetia hainanensis* in *Mouretia* (Rubiaceae). PhytoKeys 270: 273–288. https://doi.org/10.3897/phytokeys.270.180324

**DOI:** 10.3897/phytokeys.272.191461

**Published:** 2026-04-08

**Authors:** Dongxian Xu, Li Li, Buyun Zhang, Zhuqiu Song

**Affiliations:** 1 Guangdong Academy of Forestry, Guangzhou 510520, China South China Botanical Garden, Chinese Academy of Sciences Guangzhou China; 2 Key Laboratory of National Forestry and Grassland Administration on Plant Conservation and Utilization in Southern China, South China Botanical Garden, Chinese Academy of Sciences, Guangzhou 510650, China Guangdong Academy of Forestry Guangzhou China

In our recent work (Xu et al. 2026), we failed to directly cite the basionym when publishing the new combination *Mouretia hainanensis*. This omission renders the combination nomenclaturally invalid, as it is contrary to Art. 41.5 of the International Code of Nomenclature (Turland et al. 2025). We provide a formal correction below.

## Mouretia hainanensis

Taxon classificationPlantaeGentianalesRubiaceae

(H.S.Lo) Z.Q.Song & D.X.Xu comb. nov.

AB6B7167-28B9-50A9-931B-1F0C1A766169

urn:lsid:ipni.org:names:77378392-1

### Basionym.

*Mycetia hainanensis* H.S.Lo, Guihaia 11(2): 112. 1991.

### Type.

China • Hainan Province: Ledong County, Jianfeng [Jianfengling], Tianchi, in valley, 25 Apr 1982, *Q. Huang 820455* (holotype: IBSC0590313!).

## Supplementary Material

XML Treatment for Mouretia hainanensis

## Additional information

### Use of AI

No use of AI was reported.

### Author ORCIDs

Dongxian Xu https://orcid.org/0000-0002-7672-8300

Li Li https://orcid.org/0000-0002-2033-6367

Buyun Zhang https://orcid.org/0000-0003-1201-7638

Zhuqiu Song https://orcid.org/0000-0002-7763-2431

### Data availability

All of the data that support the findings of this study are available in the main text.
